# Exploiting phage receptor binding proteins to enable endolysins to kill Gram-negative bacteria

**DOI:** 10.1038/s41598-020-68983-3

**Published:** 2020-07-21

**Authors:** Athina Zampara, Martine C. Holst Sørensen, Dennis Grimon, Fabio Antenucci, Amira Ruslanovna Vitt, Valeria Bortolaia, Yves Briers, Lone Brøndsted

**Affiliations:** 1 Department of Veterinary and Animal Sciences, University of Copenhagen, Stigbøjlen 4, 1870 Frederiksberg C, Denmark; 2 Department of Biotechnology, Ghent University, Valentin Vaerwyckweg 1, 9000 Gent, Belgium; 3 National Food Institute, Technical University of Denmark, WHO Collaborating Center for Antimicrobial Resistance in Food Borne Pathogens and Genomics and European Union Reference Laboratory for Antimicrobial Resistance, Kemitorvet 204, 2800 Kongens Lyngby, Denmark

**Keywords:** Antimicrobials, Applied microbiology, Bacteriophages

## Abstract

Bacteriophage-encoded endolysins degrading the bacterial peptidoglycan are promising antibacterials for combating antibiotic-resistant bacteria. However, endolysins have limited use against Gram-negative bacteria, since the outer membrane prevents access to the peptidoglycan. Here, we present Innolysins, an innovative concept for engineering endolysins to exert antibacterial activity against Gram-negative bacteria. Innolysins combine the enzymatic activity of endolysins with the binding capacity of phage receptor binding proteins (RBPs). As proof-of-concept, we constructed 12 Innolysins by fusing phage T5 endolysin and RBP Pb5 in different configurations. One of these, Innolysin Ec6 displayed antibacterial activity against *Escherichia coli* only in the presence of Pb5 receptor FhuA, leading to 1.22 ± 0.12 log reduction in cell counts. Accordingly, other bacterial species carrying FhuA homologs such as *Shigella sonnei* and *Pseudomonas aeruginosa* were sensitive to Innolysin Ec6. To enhance the antibacterial activity, we further constructed 228 novel Innolysins by fusing 23 endolysins with Pb5. High-throughput screening allowed to select Innolysin Ec21 as the best antibacterial candidate, leading to 2.20 ± 0.09 log reduction in *E. coli* counts. Interestingly, Innolysin Ec21 also displayed bactericidal activity against *E. coli* resistant to third-generation cephalosporins, reaching a 3.31 ± 0.53 log reduction in cell counts. Overall, the Innolysin approach expands previous endolysin-engineering strategies, allowing customization of endolysins by exploiting phage RBPs to specifically target Gram-negative bacteria.

## Introduction

Development of novel antibacterials against Gram-negative bacteria is challenging because they possess an outer membrane that prevents many compounds from reaching their intracellular targets^1^. Bacteriophages (phages), viruses that infect bacteria, have naturally evolved mechanisms to overcome the outer membrane to infect their bacterial hosts^2,3^. In the first step of infection, phages bind to host cells and inject their genetic material across the outer and inner membrane of the bacterial cells into the cytoplasm^4,5^. Also, during the final stage of the lytic infection cycle, phages produce proteins within the cell, which destroy the bacterial cell wall, leading to cell lysis^6,7^. Thus, the molecular tools developed during phage evolution may be exploited to develop novel phage-based antibacterials that are able to pass the outer membrane and to kill Gram-negative bacteria.

Phages recognize their host bacteria by binding to specific surface receptors that may be outer membrane proteins, lipopolysaccharides or components of bacterial capsules, pili and flagella^8–10^. The adhesion specificity is mediated by receptor binding proteins (RBPs) that form fibers or spikes at the distal phage tail. A well-characterized RBP is the monomeric Pb5, located at the tail tip of the phage T5, which binds irreversibly to the bacterial receptor FhuA during infection of the *E. coli* host^11,12^. FhuA is an outer membrane protein that actively transports siderophore-ferrichrome and allows *E. coli* to take up iron from the environment. The crystal structure of FhuA reveals two domains, a 22-stranded anti-parallel barrel and a globular domain, known as the plug. The barrel forms a hollow channel containing eleven surface-exposed loops and is blocked by the plug in its inactive state^13,14^. Pb5 of phage T5 has been shown to have several binding targets on the FhuA, including the L4 loop and the plug of FhuA^15,16^. In addition, it was proposed that FhuA acts as an anchor for phage T5 binding to *E. coli*, subsequently allowing the tail fiber composed of Pb2 to transverse the outer membrane and inject the DNA^17^.

During the last stage of phage infection, phages produce endolysins to lyse the bacterial cells and release progeny phages. Endolysins are enzymes that, after gaining access to the periplasm, degrade the peptidoglycan, leading to cell lysis^18^. Endolysins may have different catalytic activities, depending on the bond that they target in the peptidoglycan, and are classified as glycosidases, amidases or endopeptidases. The endolysin encoded by phage T5 is a well characterized endopeptidase that hydrolyzes the bond between L-alanine and D-glutamic acid of the peptidoglycan in *E. coli*^19^. While native endolysins have been successfully applied as antibacterials against Gram-positive bacteria including *Staphylococcus aureus* and streptococci, the outer membrane of Gram-negative bacteria generally prevents access to the peptidoglycan layer^20^. To enable killing of Gram-negative pathogens using endolysins, a number of engineering strategies have been proposed. Recently it was shown that the fusion of endolysins with polycationic or amphipathic peptides enabled the engineered endolysins known as Artilysins to kill multi-drug resistant *Pseudomonas aeruginosa* and *Acinetobacter baumannii*^21,22^. Furthermore, recombinant T4 lysozyme carrying the binding domain of pesticin, targeting the outer membrane protein FyuA, killed *Yersinia* and pathogenic *E. coli* strains^23^. Similarly, fusion of the *E. coli* phage endolysin Lysep3 with the translocation and receptor-binding domain of another bacteriocin, colicin A, targeting the outer membrane protein BtuB exerted antibacterial activity against *E. coli*^24^. The translocation and receptor-binding domain of *P. aeruginosa* bacteriocin pyocin S2 (PyS2) was also recently fused with GN4 lysin to construct lysocins that could kill *P. aeruginosa*^25^. Thus, these studies have demonstrated that endolysins can be engineered to overcome the outer membrane barrier and subsequently kill Gram-negative bacteria.

Here, we expand on the concept of enabling endolysins to exert antibacterial activity against Gram-negative bacteria by exploiting the binding specificity of phage RBPs to bacterial receptors and we develop innovative RBP-endolysin hybrids (Innolysins). To provide a proof of concept, we fused the endolysin and the RBP (Pb5) of phage T5 to target *E. coli* (Innolysins Ec). Pb5 was selected among many phage RBPs due to the specific and stable binding to its cognate protein receptor, allowing the fused endolysin to exert the antibacterial activity. To expand our Innolysin concept, we constructed a library of 228 Innolysins by fusing Pb5 with 23 different endolysins in four different configurations. A high-throughput screening for the best antibacterial candidate allowed us to select an Innolysin with bactericidal activity against *E. coli* resistant to critically important antimicrobials such as third-generation cephalosporins.

## Results

### Strategy for construction of Innolysins

**Figure 1:**
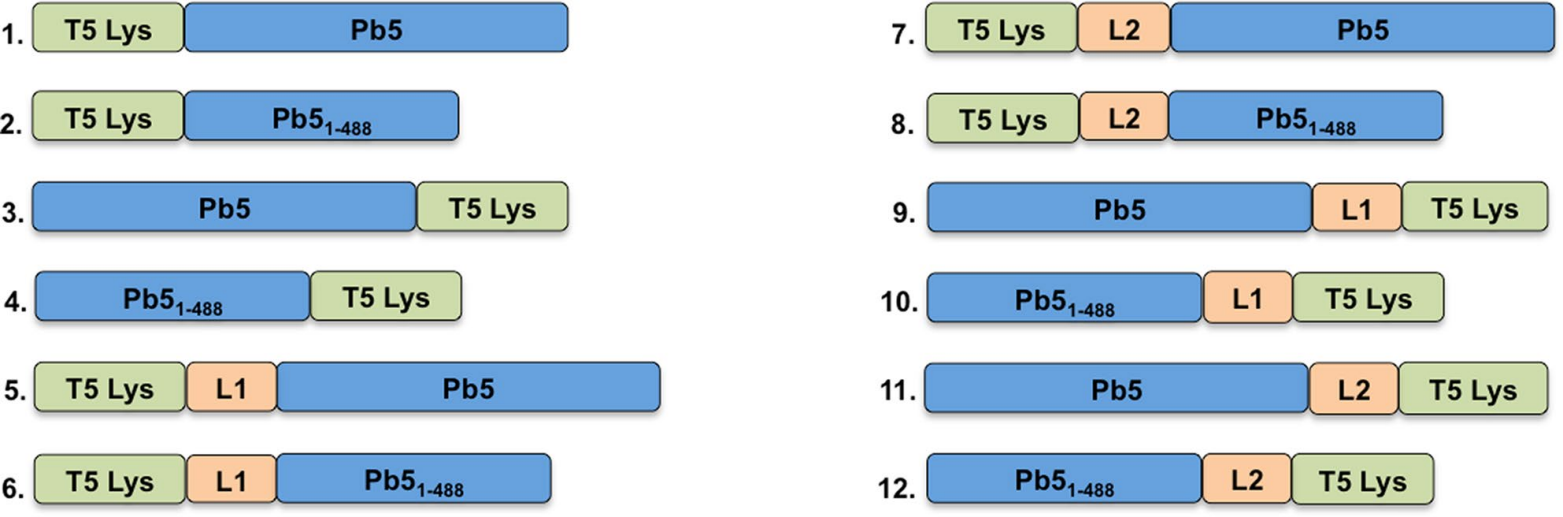
Visual representation of construction of Innolysins Ec. Phage T5 endolysin (T5 Lys) was fused with the whole phage T5 receptor binding protein, Pb5 or its binding domain (Pb5_1-488_). Fusion was conducted without linkers (Ec1 to Ec4) or with linkers (linker L1: AGAGAG or linker L2: GAGAGAGAGAGAGA) (Ec5 to Ec12) with T5 Lys located in the N-terminus (Ec1 and Ec2; Ec5 to Ec8) or in the C-terminus (Ec3 and Ec4; Ec9 to Ec12) of the engineered endolysins.

To construct *E. coli*-specific Innolysins (Innolysins Ec), we combined the RBP of phage T5 (Pb5) with the phage T5 endolysin (T5 Lys) for targeted delivery of the endolysin. The binding domain of Pb5 has previously been shown to be located in the N-terminus (488aa) of the protein^26^. To determine whether the binding domain of the Pb5 was sufficient to enable the endolysin to exert antibacterial activity, we fused T5 endolysin with both the entire Pb5 and with the Pb5 binding domain (Pb5_1-488_). We anticipated that antibacterial activity of an Innolysin Ec requires both that Pb5 is able to bind to the outer membrane protein FhuA, and that the fused phage T5 endolysin remains active to degrade the peptidoglycan. Thus, to ensure that the joined domains remain functional after fusion, we either fused them directly or added linkers in between. We used flexible linkers composed of small non-polar amino acids, glycine and alanine, providing a certain degree of flexibility of the fused domains^27^. To optimize the two-domain cooperation, we used linkers of two different sizes, L1 composed of six amino acids and L2 that consisted of 14 amino acids. In addition, to investigate the optimal orientation of the endolysin and RBP domains, we constructed the Innolysins in two directions with and without linkers. As such, a total of 12 Innolysins Ec were initially constructed (Fig. 1).

### Innolysins show muralytic activity

**Figure 2:**
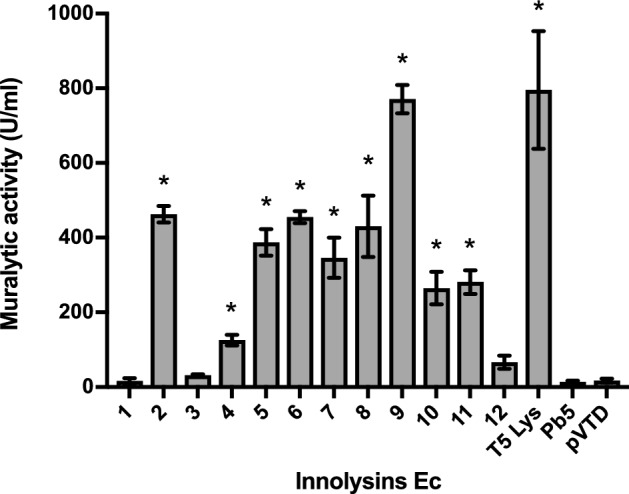
Muralytic activity of Innolysins Ec. Cleared lysates of the Innolysins Ec1-Ec12 (see Fig. 1) were screened for ability to degrade peptidoglycan of *P. aeruginosa* PAO1 compared to the activity of cleared lysates of cells carrying the empty vector, pVTD (negative control). Phage T5 endolysin (T5 Lys) and receptor binding protein (Pb5) were used as a positive and a negative control, respectively. Average muralytic activities (U/ml cleared lysate) were estimated based on triplicates. *Significant muralytic activity compared to the negative control (cleared lysate of cells carrying the empty vector, pVTD) at P < 0.05.

To demonstrate conservation of the muralytic activity of the endolysin after fusion with the RBP and a linker, the fused proteins were expressed in *E. coli* BL21 and cleared cell lysates were tested for muralytic activity. A standardized assay for analysis of the muralytic activity of endolysins against Gram-negative bacterial peptidoglycan was used. This assay is based on outer membrane permeabilized *P. aeruginosa* cells, which share a common peptidoglycan chemotype (Α1γ) with *E. coli*^28^. The majority of Innolysins (nine out of 12) were active with enzymatic activity ranging between 126–771 U/ml. Innolysin Ec9 (Pb5-L1-T5Lys) showed the highest activity per ml cleared lysate, which was similar to the muralytic activity of phage T5 endolysin alone (795 U/ml) (Fig. 2). Although expression was confirmed for all constructs, three of the Innolysins (Ec1, Ec3 and Ec12) and Pb5 alone did not show any significant activity compared to the negative control (muralytic activity of cleared cell lysates carrying the empty vector, pVTD). Yet, as expression yield was not taken into account, some of these constructs might have muralytic activity. Here, we showed that phage T5 endolysin fused with Pb5 in different configurations could maintain its muralytic activity.

### Innolysin Ec6 inhibits *E. coli* growth

**Figure 3:**
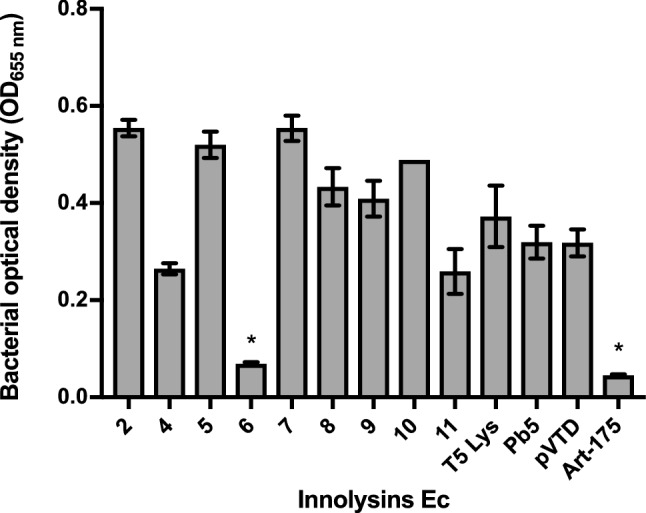
Growth of *E. coli* after treatment with Innolysins Ec. Cleared fractions of muralytically active engineered proteins (see Fig. 2) were screened for the ability to inhibit growth of *E. coli* ATCC11303. Art-175 was provided by Lysando AG^29^ and used as a positive control. Phage T5 endolysin (T5 Lys) and receptor binding protein (Pb5) were used as negative controls. Growth inhibition was detected as the absence of growth (OD_655nm_) of cells treated with an Innolysin in comparison to the negative control (growth of ATCC11303 cells treated with cleared lysate of cells carrying the empty vector, pVTD) after incubation for 18 h at 37 °C. *Significant bacterial growth inhibition at P < 0.05.

To assess whether binding of Pb5 allowed the fused endolysin to exert antibacterial activity, we screened the muralytically active Innolysins for their ability to inhibit growth of the phage T5 bacterial host *E. coli* ATCC11303. This strain was mixed with cleared lysates of cells expressing Innolysins, and bacterial growth was measured spectrophotometrically after 18 h at 37 °C (Fig. 3). Growth inhibition was determined as the lack of growth of a start inoculum treated with an Innolysin compared to growth of ATCC11303 cells treated with cleared lysates of cells carrying the empty vector, pVTD (negative control). When *E. coli* ATCC11303 was treated with Innolysin Ec6 a significant inhibitory activity was noticed, which was similar to that of Art-175, an engineered endolysin that was previously shown to have both an inhibitory and bactericidal effect against various *E. coli* isolates^29,30^. The remaining eight Innolysins, Pb5 or endolysin alone did not significantly affect the *E. coli* growth compared to the negative control (Fig. 3). Out of the 12 initially constructed Innolysins, this screening resulted in one promising antibacterial candidate that could inhibit *E. coli* growth, indicating that domains may have to be fused in a specific order and with a specific linker to acquire antibacterial activity after fusion. However, we could not exclude that a potential antibacterial candidate might not have been identified during our screen due to low protein expression yield. The active variant Ec6 was composed of T5 endolysin in the N-terminus linked by a six amino-acid linker to Pb5_1-488_ in the C-terminus. This result demonstrates that the binding domain of Pb5 is able to target the bacterial cells, allowing phage T5 endolysin to inhibit *E. coli* growth.

### Antibacterial activity of Innolysin Ec6 requires FhuA, but not TonB-provided energy

**Figure 4:**
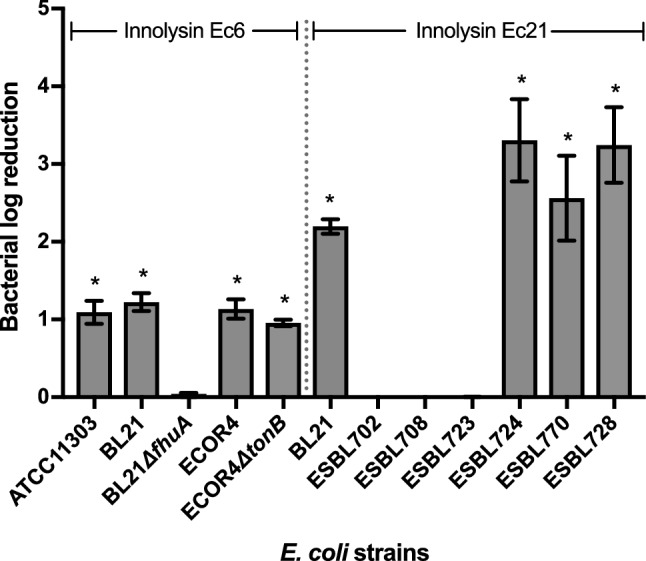
Antibacterial activity of Innolysin Ec6 and Ec21 against *E. coli*. Average logarithmic reductions in the cell counts of different *E. coli* strains, after incubation with 0.2 mg/ml either of Innolysin Ec6 or of Ec21 for 30 min at 20 °C, compared to the cells treated with 20 mM HEPES–NaOH as a negative control. The average reduction was calculated based on triplicates of three independent experiments. *Significant reduction at P < 0.05.

To determine whether FhuA is required for the antibacterial activity of Innolysin Ec6, the purified hybrid protein was tested on a *fhuA* deletion mutant of *E. coli* BL21 and compared to the wild type *E. coli* BL21 and *E. coli* ATCC11303. Bacterial cells were incubated with the purified Innolysin Ec6 at a final concentration of 0.2 mg/ml and the reduction in cell counts was compared to the cells treated with 20 mM HEPES–NaOH, as a negative control (Fig. 4). *E. coli* BL21 cell counts were reduced by 1.22 ± 0.12 log after 30 min of treatment with Innolysin and a similar decrease was noticed for *E. coli* ATCC11303 cells (1.09 ± 0.15 log reduction). In contrast, treatment of *E. coli* BL21 with either T5 endolysin or Pb5_1-488_ did not significantly reduce the cells, leading to 0.07 ± 0.06 and 0.01 ± 0.01 log reductions, respectively. No significant effect of the Innolysin was shown against *E. coli* BL21Δ*fhuA*, supporting our hypothesis that the Innolysin exerts antibacterial activity only when cells harbor the Pb5 receptor, FhuA. FhuA uptake of ferrichrome requires energy from the cytoplasmic proton motive force transduced to the outer membrane via the TonB protein, but phage T5 interacts with FhuA independent of TonB^14,31^. To determine whether activity of Innolysin Ec6 required such energy, antibacterial activity was tested against an *E. coli tonB* deletion mutant (ECOR4*ΔtonB*) and the wild type ECOR4 as a positive control (Fig. 4). Approximately 1 log reduction was demonstrated in both ECOR4*ΔtonB* and the wild type ECOR4 in cell numbers after 30 min of treatment with Innolysin Ec6 (Fig. 4), supporting that TonB was not required for Innolysin to work as an antibacterial. Our combined data demonstrate that Innolysin Ec6 requires the presence of FhuA but not TonB-provided energy to exert antibacterial activity.

### Morphological changes of cells treated with Innolysin Ec6

**Figure 5:**
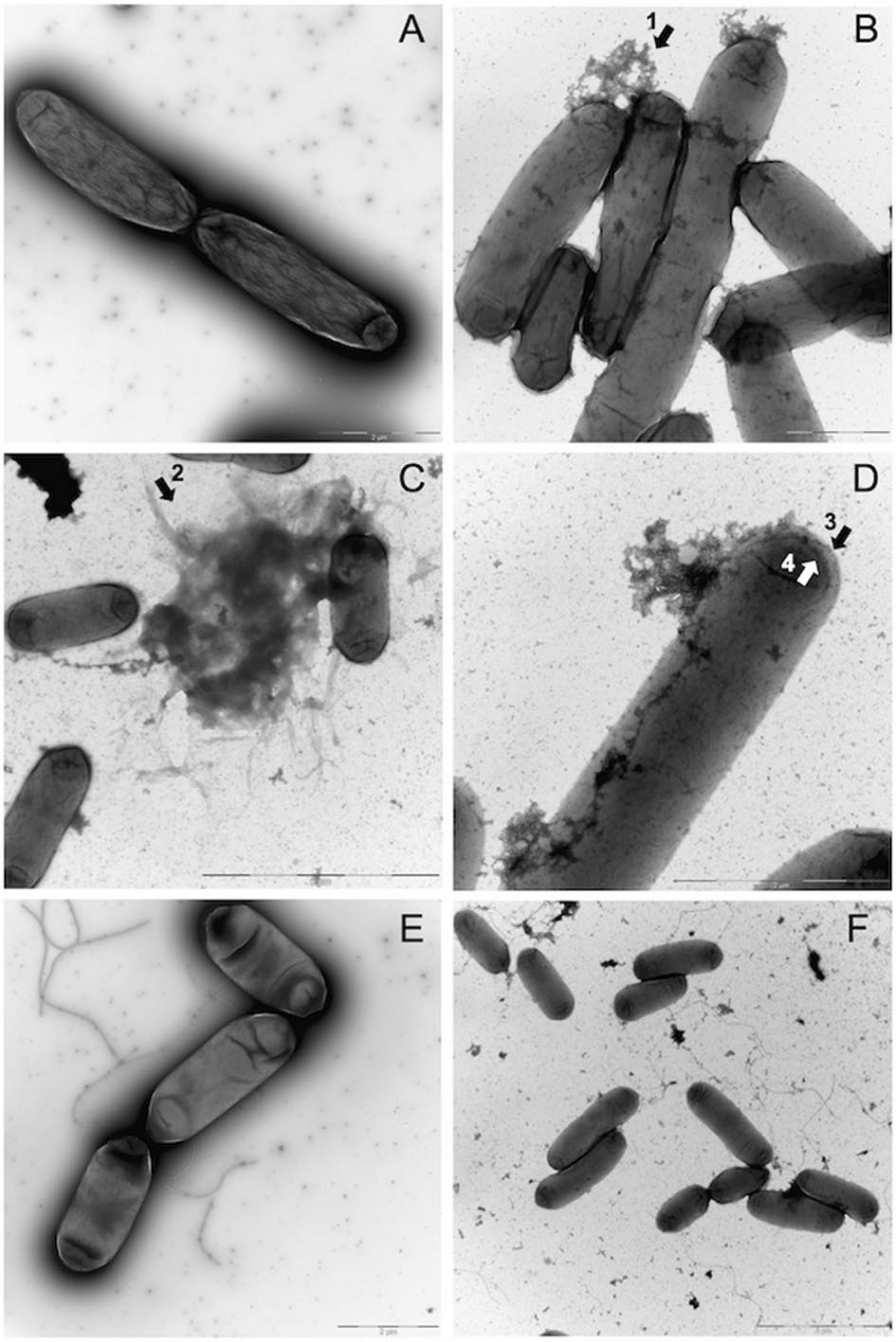
Morphological changes of *E. coli* treated with Innolysin Ec6. The effect of Innolysin Ec6 on BL21 (**B**–**D**) and BL21*ΔfhuA* (**F**) was compared to the BL21 treated with 20 mM HEPES–NaOH (**A**) and BL21*ΔfhuA* (**E**), respectively. Cytosol leakage mainly at poles (**B**, arrow 1); cell debris likely due to cell lysis (**C**, arrow 2) and increased transparency in the periphery of cells (**D**, arrow 3 & 4) were observed in the majority of BL21 cells treated with the Innolysin. Such dramatic changes were not observed in BL21*ΔfhuA* cells after incubation with Innolysin Ec6 (**F**). Images were recorded on a side-mounted Olympus Veleta (2048 × 2048 pixels) charge-coupled device camera via the iTEM software (Olympus SIS, Muenster, Germany), version 5.1 (Build 2,108), Microsoft Windows NT 5.1 (Build 2,600) Service Pack 3 2.097.151 KB total memory (https://cfim.ku.dk/equipment/electron_microscopy/cm100/iTEM_Main_Brochure.pdf).

Transmission electron microscopy (TEM) analysis was performed to determine the effects of Innolysin Ec6 on cell morphology and viability. The morphology of both *E. coli* BL21 and *E. coli* BL21*ΔfhuA* was visualized after incubation with 0.2 mg/ml Innolysin Ec6 for 15 min and compared to cells treated with 20 mM HEPES–NaOH (pH 7.4) as a negative control (Fig. 5). Almost all cells treated with HEPES–NaOH were intact with normal cell envelope morphology. In contrast, treatment of *E. coli* BL21 with Innolysin Ec6 led to cell integrity damage in the majority of cells with cytosol leakage occurring mainly at the poles. Furthermore, cell debris could be detected possibly due to cell lysis. This dramatic effect on cell morphology was not observed on *E. coli* BL21*ΔfhuA* treated with Innolysin Ec6, where only a few damaged cells were noticed compared to the cells treated with HEPES–NaOH. These observations demonstrate that Innolysin Ec6 acts rapidly by interfering with membrane integrity and reconfirms that the presence of FhuA is essential for Innolysin Ec6 to exert high antibacterial activity.

### Innolysin Ec6 targets FhuA homologs of species other than *E. coli*

**Figure 6:**
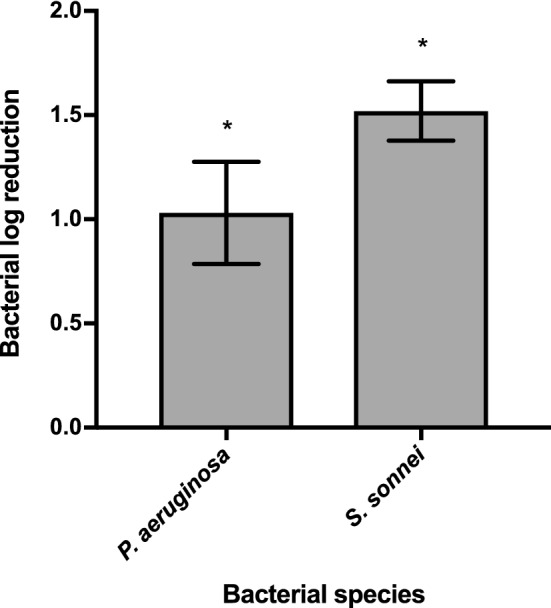
Antibacterial spectrum of Innolysin Ec6. Average logarithmic reductions of different species in cell numbers, after incubation with 0.2 mg/ml Innolysin Ec6 for 30 min at 20 °C, compared to the cells treated with 20 mM HEPES–NaOH as a negative control. The data represent the average of three biological triplicates with three technical replicates each. * Significant reduction at P < 0.05.

To investigate whether the constructed Innolysin Ec6 could target FhuA homologs in other species, we tested the antibacterial activity of the purified Innolysin against *Shigella sonnei* and *Pseudomonas aeruginosa* (Fig. 6). These bacteria carry FhuA homologs with identity to the FhuA of *E. coli* BL21 ranging between 22.6 and 99.6% (Supplementary Table S1). The Innolysin displayed antibacterial activity against *S. sonnei* (99.6% FhuA identity) and *P. aeruginosa* PAO1 (less than 39% of overall FhuA identity), leading to average log reductions in cell number of 1.52 ± 0.14 and 1.03 ± 0.25, respectively. To give support that the antibacterial activity of Innolysin Ec6 against the tested strains is specific for the Innolysin, we further tested the antibacterial activity of either T5 endolysin or the Pb5 _1–488_. Treatment with T5 endolysin did not significantly reduce the cell counts of either *P. aeruginosa* (0.08 ± 0.02 log reduction) or *S. sonnei*, (0.09 ± 0.04 log reduction). Similarly, Pb5_1-488_ did not display significant antibacterial activity against both strains, leading to 0.04 ± 0.03 and 0.07 ± 0.05 log reduction of the cells, respectively. Therefore, we conclude that Innolysin can target FhuA homologs in species other than *E. coli* and exert antibacterial activity.

### Enhanced Innolysins with activity against *E. coli* resistant to third-generation cephalosporins

To further explore the Innolysin concept, we constructed a library comprising 228 novel Innolysins by fusing one out of 23 different endolysins (Supplementary Table S2) with Pb5 or Pb5_1-488_ in four configurations (as described in the supplementary method and Fig. S1). To identify Innolysins with high antibacterial activity, we initially used a high-throughput growth inhibitory assay on *E. coli* BL21 and screened the library in a redundant way by analyzing 380 variants (i.e., 95 variants from each configuration). We selected 12 Innolysins showing the strongest growth inhibition for each configuration (Supplementary Table S3 and S4). Of these 48 Innolysins, 32 were confirmed to be muralytically active (Supplementary Fig. S2) and could significantly inhibit the *E. coli* BL21 growth based on three independent experiments (Supplementary Fig. S3). Interestingly, Innolysins belonging to all four configurations could inhibit *E. coli* BL21 growth with Innolysin Ec21 demonstrating the highest growth inhibition effect. Innolysin Ec21 was composed of endolysin LysEC8 fused in its C-terminus with Pb5_1-488_ by a flexible linker (Supplementary Table S4). To assess whether LysEc8 alone could exert antibacterial activity, the killing efficiency of LysEc8 was tested against *E. coli* BL21. No significant effect on the cell numbers was detected with log reduction reaching to 0.08 ± 0.07. In contrast, application of purified Innolysin Ec21 (0.2 mg/ml) on *E. coli* BL21 led to 2.20 ± 0.09 log reduction in cell numbers, thus increasing the antibacterial activity compared to Innolysin Ec6 that led to 1.22 ± 0.12 log reduction (Fig. 4). To determine whether Innolysin Ec21 was also effective against antibiotic-resistant *E. coli* strains, the antibacterial activity was tested against six *E. coli* resistant to third-generation cephalosporins isolated from production animals and meat (Supplementary Table S5). Interestingly, three out of the six of such *E. coli* strains were sensitive to Innolysin Ec21 with the maximum bactericidal activity reaching to 3.31 ± 0.53 log reduction in cell number (Fig. 4). In contrast, no reduction of cell counts was observed when the remaining three *E. coli* strains were treated with Innolysin Ec21, displaying resistance. Therefore, our results indicate a variable sensitivity of antibiotic-resistant *E. coli* resistant to third-generation cephalosporins to Innolysin Ec21.

**Figure 7:**
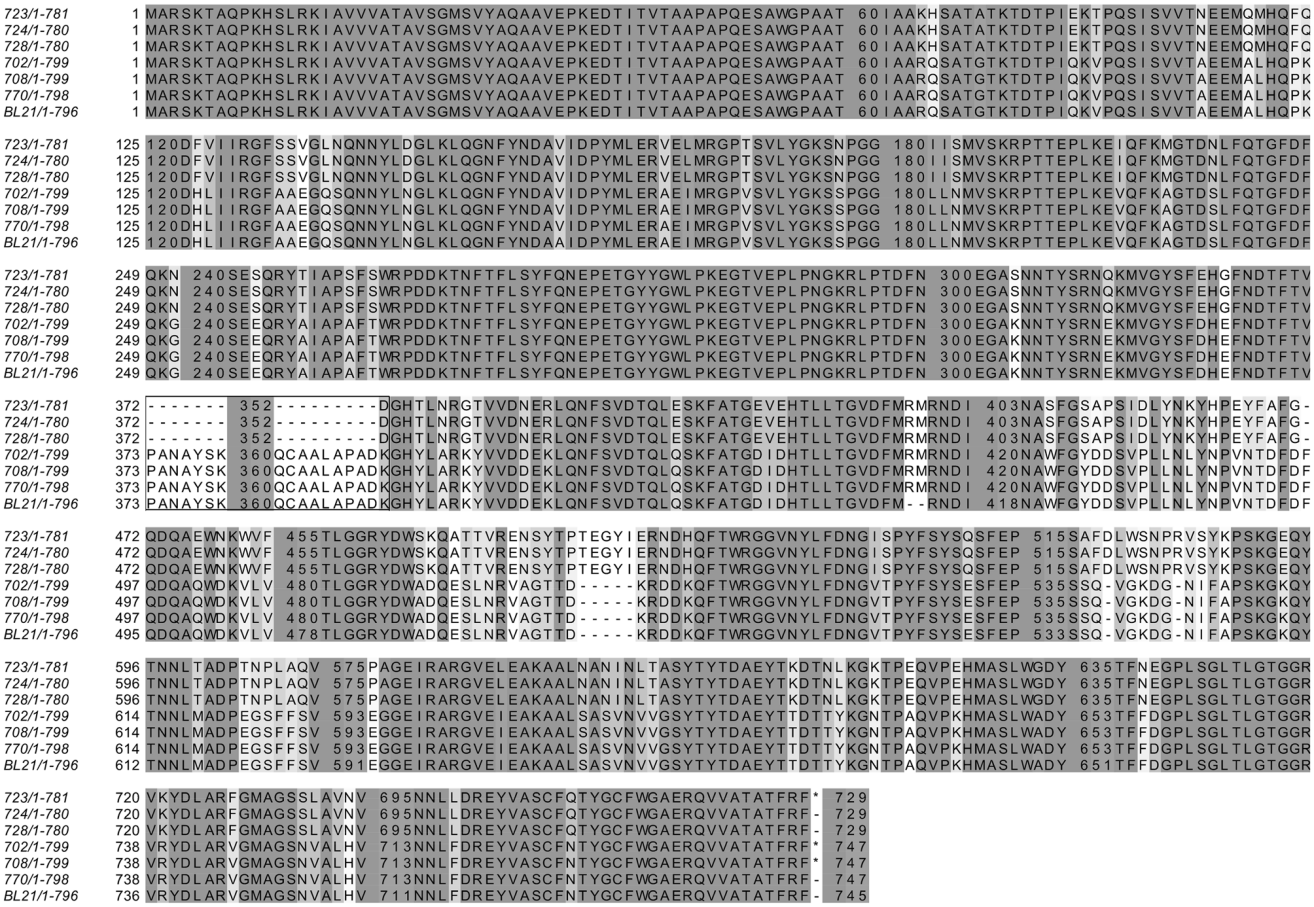
Alignment of *E. coli* BL21 FhuA with homologous proteins of *E. coli* resistant to third-generation cephalosporins. *E. coli* BL21 FhuA (WP_000124402.1) was aligned to either FhuA or Ferrichrome Iron Receptors (FIR) of ESBL-producing *E. coli* strains (702: FIR (ESC_RA7174AA_AS_04180); 708: FIR (ESC_RA7666AA_AS_01170); 723: FhuA (ESC_RA7193AA_AS_00052); 724: FhuA (ESC_RA7194AA_AS_03122); 728: FhuA (ESC_RA7198AA_AS_01051); 770: FIR (ESC_RA7701AA_AS_01976)). Amino acids are colored based on the level of conservation with transition from light to dark grey to represent increasing conservation. Conservation with no threshold was used when we used the Jalview sequence alignment tool. The box highlights the alignment in the region responsible for the formation of the surface-exposed part of loop L4 in *E. coli* BL21.

To understand the variability in antibacterial spectrum of the *E. coli* strains tested, we investigated whether the sensitivity pattern correlates with differences in the FhuA protein sequence, potentially affecting the interaction and activity of Innolysin Ec21. Alignment of the FhuA protein sequences showed that Innolysin sensitivity was not correlated with the presence of specific loops of the FhuA barrel, including the L4 loop previously shown to be one of the binding targets of phage T5 Pb5^15,17^. Some sensitive strains even lacked the amino acids responsible for the formation of L4 loop in FhuA (Fig. 7), suggesting that the L4 loop is not required for the antibacterial activity of Pb5-based Innolysins. In addition, sequence variation was observed in other FhuA regions between strains, but with no correlation to the sensitivity profile. Thus, variations in FhuA cannot explain the Innolysin Ec21 killing spectrum of antibiotic-resistant *E. coli*. Overall, by shuffling endolysin components we enhanced the antibacterial activity of Innolysins and showed that Innolysins can be used as antibacterials to kill at least a subset of antibiotic-resistant *E. coli*.

## Discussion

Phages have developed unique and complex mechanisms to infect and lyse bacteria. In the first stage of infection, phages utilize RBPs to target specific bacterial host receptors on the cell surface, whereas at later stages phage endolysins degrade peptidoglycan, inducing lysis and progeny release. Here, we exploited the binding capacity of a phage RBPs to enable an endolysin to exert antibacterial activity. As proof of concept, we used the phage T5 endolysin and its RBP Pb5 and constructed 12 Innolysins by fusing the endolysin to the whole Pb5 or the binding domain of Pb5 in different orientations, with or without linkers. The majority of the novel Innolysins maintained their muralytic activity and Innolysin Ec6 also reduced the *E. coli* cell viability by approximately 1 log. To improve the antibacterial activity of Innolysins, we redundantly screened a library of 228 novel Innolysins each consisting of one out of 23 different endolysins fused with Pb5 in four distinct configurations. Growth inhibition was obtained for Innolysins from all four classes, indicating that Pb5 could be fused with different endolysins in several configurations and still could enable the fused endolysins to exert antibacterial activity. Interestingly, high-throughput screening for the best antibacterial candidate allowed the identification of Innolysin Ec21, that increased the reduction of *E. coli* cells compared to Innolysin Ec6, reaching to approximately 2 log. The two Innolysins share the same configuration (endolysin-Linker 2-Pb5_1-488_), but differ in the endolysin component, as Innolysin Ec6 and Ec21 contain phage T5 endolysin and LysEC8, respectively.

Here we showed that neither the cell viability nor the morphology of BL21Δ*fhuA* cells was changed after application of Innolysin Ec6, indicating that antibacterial activity of the Innolysin is dependent on the presence of the phage T5 receptor, FhuA^11,12^. Since FhuA functions as a transport channel for uptake of ferrichrome, it is tempting to speculate that an Innolysin might be transported through the channel. Yet, it has previously been shown that Pb5 binding to FhuA did not open the channel^11,15^. Furthermore, our results demonstrate that Innolysin Ec6 activity is independent of energy provided by TonB, which is needed for the opening of the channel. Therefore, binding of an Innolysin might not provide the conformational changes, leading to the opening of FhuA channel. In addition, the size of the unplugged channel (2.5 nm)^13,14^ appeared to be too narrow for Innolysin Ec6 (67.62 kDa) to pass^32^, similarly to what was previously suggested for a hybrid lysin consisting of the phage T4 lysozyme and the binding domain of pesticin targeting FyuA^19^. Thus, we hypothesize that Innolysins might overcome the outer membrane barrier by other means than passing through the FhuA channel.

One possible way that Innolysins could access the peptidoglycan might be by interfering with the membrane integrity. FhuA has been proposed to function as an anchor for phage T5 by an irreversible binding of Pb5 to the FhuA receptor^15,17^. We combined Pb5 with phage T5 endolysin, which is a globular endolysin carrying a single enzymatically active domain (EAD) with limited intrinsic antibacterial activity^33^. By irreversible binding to FhuA^11^, Pb5 might bring the Innolysin in close proximity to the outer membrane. Therefore, the Innolysin could interfere with phosphates in the outer membrane lipopolysaccharides and displace the stabilizing Mg^2+^/Ca^2+^ ions due to the basic isoelectric point of phage T5 endolysin (7.91), thus destabilizing the ionic forces in the outer membrane^34^. Outer membrane interference has also been described for Artilysins, endolysins engineered with polycationic or amphipathic peptides that destabilize the outer membrane and target the endolysins to peptidoglycan^21^. Similar to polycationic peptides, the positively charged N-terminal extension of Thermus phage 2,631 endolysin has been shown to be crucial for the enzyme to pass through the outer membrane and exert a strong antibacterial activity against Gram-negative bacteria^35,36^. This mode of action could also explain how substituting the T5 endolysin by other endolysin components with higher pI resulted in an increase of the antibacterial activity of Innolysin Ec21. LysEC8 endolysin, the endolysin component in Innolysin Ec21, displays a pI of 9.16 exceeding the T5 endolysin pI (7.91). This difference might influence the distribution of ions in the surrounding interfacial region, thereby destabilizing outer membrane ionic interactions more efficiently. Interestingly, we noticed that Innolysin Ec6 caused cytosol leakage mainly from the bacterial poles of the wild type *E. coli* carrying the FhuA. It has been shown that several phages preferentially adsorb at the bacterial poles, including *E. coli* phage φ80 that uses FhuA as receptor^37^. Therefore, Innolysins may bind to the bacterial surface in a similar manner as phages and interfere locally with the outer membrane integrity to allow the fused endolysins to target peptidoglycan. However, further insights in the exact mechanism by which the RBPs enable the fused endolysins to exert antibacterial activity are needed to clarify the mode of Innolysins action.

Interestingly, Innolysin Ec21 demonstrated bactericidal activity against three out of six tested *E coli* strains resistant to third-generation cephalosporins with killing efficiency reaching to 3.31 ± 0.53 log reduction in only 30 min. This diverse killing spectrum of Innolysin Ec21 could not be explained by differences in the binding ability of the Innolysin to FhuA since killing abilities were not correlated with differences in the FhuA protein sequence, including the L4 loop that is one of the binding targets of phage T5^31,38^. Therefore, other mechanisms such as receptor masking by lipopolysaccharides (LPS) or downregulation of receptor expression^39,40^ could play a role in Innolysin resistance. Furthermore, if Innolysins indeed interfere with the ionic interactions, differences in LPS composition could affect membrane integrity and could explain the variation in sensitivity to Innolysins similarly. Therefore, the killing efficiency of an Innolysin might be affected not only by the endolysin component but also by the outer membrane composition of the specific strain.

In this study, we used the binding specificity of the phage T5 RBP to its cognate FhuA receptor to enable an endolysin to exert antibacterial activity. FhuA is conserved in bacterial species, including *Escherichia* and *Shigella*, and is structurally homologous to other TonB-dependent outer membrane receptors involved in bacterial iron uptake^41^. Our results demonstrate that Innolysin Ec6, similar to bacteriocin-derived fusions with endolysins, can target isolates of bacterial species other than *Escherichia coli*, including *Shigella sonnei* and *Pseudomonas aeruginosa*. As opposed to some bacteriocins and traditional antibiotics targeting outer membrane proteins^42^, Innolysins exert antibacterial activity against Gram-negative bacteria independent of the energy provided by TonB. These results give support to the potential of using Innolysins against non-actively growing bacteria. Similar to antibiotics, it is expected that bacterial resistance to Innolysins may emerge, putatively by mutations on the binding targets of Innolysins. Yet, some mutations of receptors targeted by phages are known to affect the bacterial virulence. In the case of Innolysins Ec, mutations in FhuA may not be critical for the bacterial virulence because *E. coli* use at least seven iron acquisition systems^43^. However, having provided the proof of concept will now allow us to use the vast diversity of phage RBPs to design Innolysins able to target outer membrane structures that constitute virulence determinants such as OmpX in *Escherichia coli* and Ail in *Yersinia pesti*s^44,45^. In summary, we have demonstrated a novel antibacterial concept and have shown the potential for optimization of Innolysins activity by shuffling. In future, this concept may be further expanded to target other Gram-negative bacteria exploiting phage RBPs binding specificity and the vast diversity of bacterial receptors.

## Materials and methods

### Bacterial strains

*E. coli* BL21(DE3) CodonPlus-RIL (Agilent Technologies) was used for expression of recombinant proteins. Inhibition assays were conducted with *E. coli* ATCC11303 (Leibniz Institute) and *E. coli* BL21(DE3) (Agilent Technologies). *E. coli* ECOR4 (STEC Center at Michigan State University), *Shigella sonnei*^46^ and *Pseudomonas aeruginosa* PAO1^47^ were also used for antibacterial activity of Innolysin Ec6. Deletions of the *fhuA* and *tonB* genes on the chromosomes of *E. coli* BL21(DE3) and *E. coli* ECOR4, respectively, were previously generated by the Lambda red recombination system as described before^48^. Antibacterial activity of Innolysin Ec21 was tested against six *E. coli* strains resistant to third-generation cephalosporins collected during surveillance of antibiotic resistance in indicator *E. coli* from production animals and food in Denmark. These *E. coli* strains exhibit an AmpC phenotype due to promoter mutations leading to upregulation of the chromosomal *ampC* and belong to different sequence types (ST-types) by Multi Locus Sequence Typing (Supplementary Table S5).

### Cloning of constructs

Linker L1 (AGAGAG) or linker L2 (GAGAGAGAGAGAGA) were used for joining the coding sequences of Pb5 and the endolysins, whereas a His-tag was fused to the C-terminus of all constructs. Cloning of DNA fragments encoding endolysins, linkers and His-tag and their assembly to Innolysin encoding sequences was conducted as described elsewhere^49,50^. In brief, genomic material was purified from phage T5 (Leibniz Institute) and used as a template for amplification (*Pfu* polymerase; Thermo Fisher Scientific, Waltham, MA) of the genes encoding T5 endolysin (YP_006868.1) or Pb5 (YP_006985.1) with specific primers (Supplementary Table S6). The amplified DNA fragments were separately cloned into the pVTE vector by conducting 30 cycles of restriction with 10 U SapI type IIs restriction enzyme (37 °C, 2 min) and ligation with 3U T4 DNA ligase (16 °C, 3 min), followed by transformation of *E. coli* TOP10. Transformed cells were selected on LB (Lysogeny Broth) agar plates in the presence of ampicillin (100 μg/ml) and supplemented with 5% sucrose as a positive selection for recombinants. Subsequently, these pVTE plasmids were extracted and used for assembly of the different DNA fragments (50 ng/µl of each pVTE vector) into the concatenated sequence encoding an Innolysin. This assembly and the insertion into destination vector pVTD was done using 10U BsaI and 3U T4 DNA ligase and the same cyclic reaction as above. The assembly reaction mixture was used for transformation of *E. coli* BL21(DE3)-CodonPlus RIL cells by heat shock. Transformant cells were plated on LB agar plates supplemented with kanamycin (100 μg/ml) and chloramphenicol (50 μg/ml).

### Protein expression and purification

For the library screening, 95 single colonies were randomly picked up from each configuration for downstream analysis. Freshly transformed colonies were resuspended in 500 μl of auto-induction medium (93% ZY medium, 0.05% 2 M MgSO_4_, 2% 50 × 5,052, 5% 20 × NPS) in a 96 deep-well plate and incubated at 37 °C for 5 h, followed by 16 °C for 40 h, both at 900 rpm. Cells were spun down by centrifugation (3,200 × *g*, 30 min, 4 °C) and the supernatants were removed. Cell pellets were lysed by exposure to chloroform vapor, by placing the deep-well plate upside-down in a glass petri dish containing chloroform-saturated filters for 2 h. Cell lysates were resuspended in 20 mM HEPES–NaOH (pH 7.4), and 1 U DNase I, followed by incubation (100 × rpm, 1 h, 30 °C). The lysates were investigated by SDS-PAGE analysis to confirm the protein expression, but protein yield could not be accurately quantified. Insoluble fractions of cell lysates were removed by centrifugation (3,200 × *g* for 30 min, at 4 °C) and the cleared lysates were screened for both muralytic and inhibitory activities.

For large-scale expression of the Innolysin Ec6 and Ec21, a 1 L expression culture (LB medium) was induced with 1 mM isopropyl-beta-D-thiogalactopyranoside (Thermo Fisher Scientific) in the mid-logarithmic phase (OD_600_ = 0.6). Incubation of the culture was performed at 16 °C for 18 h at 120 rpm. Cells were harvested by centrifugation (8,000 × *g*, 10 min, 4 °C) and the cell pellet was resuspended in 10 ml of lysis buffer (20 mM NaH_2_PO_4_-NaOH, 0.5 M NaCl, 50 mM imidazole, pH 7.4), followed by sonication (Bandelin Sonopul HD 2070 homogeniser) with 10 bursts of 30 s (amplitude of 50%) with 30 s intervals. Protein lysate was double-filtered using filters with pore size of 0.22 μm. His GraviTrap™ gravity flow columns (GE Healthcare) were used for His-tagged protein purification according to the manufacturer’s instructions. Buffer exchange was performed against 20 mM HEPES–NaOH (pH 7.4) by using Amicon^R^ Ultra—4 centrifugal filters with 50 kDa cutoff (Merck Millipore) and protein concentration was measured by Qubit™ Protein Assay Kit (Q33211) with a Qubit 2.0 Fluorometer (Invitrogen, Q32866).

### Muralytic assay

Analysis of the muralytic activity was conducted as described before^21,51^ using outer membrane permeabilized *P. aeruginosa* PAO1 cells as substrate. Briefly, exponentially growing cells (OD_600_ = 0.6) were harvested by centrifugation (3,200 × *g*, 30 min, 4 °C) and permeabilized by resuspension in chloroform-saturated 0.05 M Tris–HCl (pH 7.7) and gentle shaking for 45 min. To remove chloroform traces, cells were washed twice with phosphate-buffered saline (PBS, pH 7.4) and further concentrated to OD_600_ = 1.5. 30 μl of the cleared lysates was added on top of 270 μl of the substrate. Cleared lysates of cells expressing the phage T5 endolysin or carrying an empty vector (pVTD) were used as positive and negative controls, respectively. Turbidities were measured spectrophotometrically at 655 nm every 30 s for one hour by a Microplate Reader 680 system (Bio-Rad). Muralytic activities were calculated by a previously described standardized method^52^.

### Growth inhibition assay

*E. coli* cells were used for the growth inhibition assay. Overnight cell cultures prepared in Mueller Hinton (MH) broth were adjusted to OD_600_ = 0.1 in 2×MH and further 100-fold diluted in 2×MH. 50 µl of the cell suspension was mixed with 50 μl of the soluble lysate fraction. Art-175 (0.1 mg/ml) and the soluble lysate fraction of cells carrying an empty vector pVTD were mixed with cells as positive and negative controls, respectively. Endpoint measurement was performed spectrophotometrically at 655 nm after exactly 18 h incubation at 37 °C. All experiments were done in biological triplicate.

### Antibacterial assay

Overnight cultures of *E. coli* strains were subcultured in LB and supplemented with 2 μg/ ml cefotaxime in case of the *E. coli* strains resistant to third-generation cephalosporins. Cells were grown exponentially (OD_600_ = 0.6) and 100-fold diluted in 20 mM HEPES–NaOH (pH 7.4). 100 μl of the diluted cell suspensions were mixed with 100 μl of the pure Innolysin at a final concentration of 0.2 mg/ml. 100 μl of 20 mM HEPES–NaOH (pH 7.4) was added to the cells as a negative control. The samples were incubated for 30 min at 20 °C, and appropriate cell dilutions were plated in triplicate on LB agar plates, with 2 μg/ ml cefotaxime in case of *E. coli* strains resistant to third-generation cephalosporins. After overnight incubation at 37 °C, colony forming units (CFU) were counted and cell concentrations (CFU/ml) were calculated. Experiments were conducted three independent times. The antibacterial activity was determined based on the difference of the average logarithmic cell concentrations of the Innolysin treated samples compared to the negative control.

### Transmission electron microscopy

Exponentially growing cells (1 ml) were harvested by centrifugation (10,000 rpm, 5 min) and resuspended in 200 μl of 20 mM HEPES–NaOH (pH 7.4). This washing procedure was repeated 3 times, after which the cell pellets were resuspended in either 50 μl of HEPES buffer or the Innolysin Ec6 and incubated for 15 min at 20 °C. Samples were negatively stained with 2% uranyl-acetate on glow-discharged continuous carbon-coated 300-mesh copper grids (EM Resolutions Ltd). Transmission electron microscopy was performed on a Philips CM100 (Tungsten emitter) electron microscope operating at 100 kV. Images were recorded on a side-mounted Olympus Veleta (2048 × 2048 pixels) charge-coupled device camera via the iTEM software (Olympus SIS, Muenster, Germany).

### Bioinformatic analysis

To identify FhuA homologs, we used the FhuA protein sequence of *E. coli* BL21 (WP_000124402.1*) *to search homologous proteins in the National Center for Biotechnology Information (NCBI) genome database through BLASTP^53^. Homologous proteins with different levels of identity in species other than *E. coli* were selected and aligned by the multiple sequence alignment tool Clustal Omega^54^, through which the percent identity was obtained (Supplementary Table S1). Clustal Omega was also used to align FhuA or Ferrichrome Iron Receptor (FIR) protein sequences obtained by in silico analysis of ESBL-producing *E. coli* strains (702: FIR (ESC_RA7174AA_AS_04180); 708: FIR (ESC_RA7666AA_AS_01170); 723: FhuA (ESC_RA7193AA_AS_00052); 724: FhuA (ESC_RA7194AA_AS_03122); 728: FhuA (ESC_RA7198AA_AS_01051); 770: FIR (ESC_RA7701AA_AS_01976), using the protein sequence of *E. coli* BL21 FhuA (WP_000124402.1) as a reference. To further illustrate the conserved regions of the proteins (Fig. 7) we used the Jalview sequence alignment tool^55^.

### Statistical analysis

Analysis of the data was conducted by using GraphPad Prism 7 software (Version 7.0d). For muralytic assays, the activity of each cleared lysate of cells expressing Innolysins was tested in triplicate and means of activity was compared with the average activity of the cleared lysates of cells carrying the empty vector. The significance of the difference in muralytic activity was assessed with Unpaired-Samples t-test using 95% confidence interval for the mean difference. The same software and also the same statistical test was used to analyze results from the growth inhibition assays, using the optical density (OD_655_) of the bacterial growth after incubation with the cleared lysate of each protein compared to the optical density of cells grown after treatment with the cleared lysate of cells carrying the empty vector. For the antibacterial assay, all bacterial counts were converted to log-scale and means, and standard deviations were calculated afterwards. Antibacterial activity was tested in triplicate in three independent experiments. Decimal reductions of cells were calculated by the difference between the average logarithmic concentrations of cells treated with Innolysin and cells treated with 20 mM HEPES–NaOH (pH 7.4) as a negative control. The significance of the decimal reductions of cells was assessed with Paired-Samples t-test using 95% confidence interval percentage.

## Supplementary information

Supplementary file1

## References

[1] Blair JM, Webber MA, Baylay AJ, Ogbolu DO, Piddock LJ (2015). Molecular mechanisms of antibiotic resistance. Nat. Rev. Microbiol..

[2] Chaturongakul S, Ounjai P (2014). Phage–host interplay: Examples from tailed phages and Gram-negative bacterial pathogens. Front. Microbiol..

[3] Samson JE, Magadan AH, Sabri M, Moineau S (2013). Revenge of the phages: defeating bacterial defences. Nat. Rev. Micro..

[4] Nobrega FL (2018). Targeting mechanisms of tailed bacteriophages. Nat. Rev. Microbiol..

[5] Grayson P, Molineux IJ (2007). Is phage DNA ‘injected’ into cells—biologists and physicists can agree. Curr. Opin. Microbiol..

[6] 6Cahill, J. & Young, R. in *Adv. Virus Res.* Vol. 103 (eds Margaret Kielian, Thomas C. Mettenleiter, & Marilyn J. Roossinck) 33–70 (Academic Press, 2019).

[7] Young R (2014). Phage lysis: Three steps, three choices, one outcome. J. Microbiol..

[8] Rakhuba DV, Kolomiets EI, Dey ES, Novik GI (2010). Bacteriophage receptors, mechanisms of phage adsorption and penetration into host cell. Pol. J. Microbiol..

[9] Letarov AV, Kulikov EE (2017). Adsorption of bacteriophages on bacterial cells. Biochemistry (Mosc.).

[10] Bertozzi Silva J, Storms Z, Sauvageau D (2016). Host receptors for bacteriophage adsorption. FEMS Microbiol. Lett..

[11] Plancon L (2002). Characterization of a high-affinity complex between the bacterial outer membrane protein FhuA and the phage T5 protein pb5. J. Mol. Biol..

[12] Mondigler M, Vögele RT, Heller KJ (1995). Overproduced and purified receptor binding protein pb5 of bacteriophage T5 binds to the T5 receptor protein FhuA. FEMS Microbiol. Lett..

[13] Ferguson AD, Hofmann E, Coulton JW, Diederichs K, Welte W (1998). Siderophore-mediated iron transport: crystal structure of FhuA with bound lipopolysaccharide. Science.

[14] Locher KP (1998). Transmembrane signaling across the ligand-gated FhuA receptor: Crystal structures of free and ferrichrome-bound states reveal allosteric changes. Cell.

[15] Flayhan A, Wien F, Paternostre M, Boulanger P, Breyton C (2012). New insights into pb5, the receptor binding protein of bacteriophage T5, and its interaction with its *Escherichia coli* receptor FhuA. Biochimie.

[16] Endriß F, Braun V (2004). Loop deletions indicate regions important for FhuA transport and receptor functions in *Escherichia coli*. J. Bacteriol..

[17] Böhm J (2001). FhuA-mediated phage genome transfer into liposomes: A cryo-electron tomography study. Curr. Biol..

[18] Oliveira H (2013). Molecular aspects and comparative genomics of bacteriophage endolysins. J. Virol..

[19] Mikoulinskaia GV (2009). Identification and characterization of the metal ion-dependent l-alanoyl-d-glutamate peptidase encoded by bacteriophage T5. FEBS J..

[20] Schmelcher M, Loessner MJ (2016). Bacteriophage endolysins: Applications for food safety. Curr. Opin. Biotechnol..

[21] Briers Y (2014). Engineered endolysin-based “Artilysins” to combat multidrug-resistant gram-negative pathogens. MBio.

[22] Defraine V (2016). Efficacy of Artilysin® Art-175 against resistant and persistent *Acinetobacter baumannii*. Antimicrob. Agents Chemother..

[23] Lukacik P (2012). Structural engineering of a phage lysin that targets Gram-negative pathogens. Proc. Natl. Acad. Sci..

[24] Yan G (2017). The N-terminal and central domain of colicin A enables phage lysin to lyse *Escherichia coli* extracellularly. Antonie Van Leeuwenhoek.

[25] Heselpoth RD, Euler CW, Schuch R (2019). Lysocins: bioengineered antimicrobials that deliver lysins across the outer membrane of gram-negative bacteria. Antimicrob. Agents Chemother..

[26] Mondigler M, Holz T, Heller KJ (1996). Identification of the receptor-binding regions of pb5 proteins of bacteriophages T5 and BF23. Virology.

[27] Argos P (1990). An investigation of oligopeptides linking domains in protein tertiary structures and possible candidates for general gene fusion. J. Mol. Biol..

[28] Schleifer KH, Kandler O (1972). Peptidoglycan types of bacterial cell walls and their taxonomic implications. Bacteriol. Rev..

[29] Briers Y (2014). Art-175 is a highly efficient antibacterial against multidrug-resistant strains and persisters of *Pseudomonas aeruginosa*. Antimicrob. Agents Chemother..

[30] Schirmeier E (2017). Inhibitory and bactericidal effect of Artilysin^**®**^ Art-175 against colistin-resistant mcr-1-positive *Escherichia coli* isolates. Int. J. Antimicrob. Agents.

[31] Killmann H, Videnov G, Jung G, Schwarz H, Braun V (1995). Identification of receptor binding sites by competitive peptide mapping: phages T1, T5, and phi 80 and colicin M bind to the gating loop of FhuA. J. Bacteriol..

[32] Erickson HP (2009). Size and shape of protein molecules at the nanometer level determined by sedimentation, gel filtration, and electron microscopy. Biol. Proced. Online.

[33] Shavrina MS (2016). In vitro study of the antibacterial effect of the bacteriophage T5 thermostable endolysin on *Escherichia coli* cells. J. Appl. Microbiol..

[34] Gerstmans H, Criel B, Briers Y (2017). Synthetic biology of modular endolysins. Biotechnol. Adv..

[35] Plotka M (2019). Structure and function of the Ts2631 endolysin of Thermus scotoductus phage vB_Tsc2631 with unique N-terminal extension used for peptidoglycan binding. Sci. Rep..

[36] Plotka M, Kapusta M, Dorawa S, Kaczorowska A-K, Kaczorowski T (2019). Ts2631 endolysin from the extremophilic thermus scotoductus bacteriophage vB_Tsc2631 as an antimicrobial agent against gram-negative multidrug-resistant bacteria. Viruses.

[37] Edgar R (2008). Bacteriophage infection is targeted to cellular poles. Mol. Microbiol..

[38] Bonhivers M, Ghazi A, Boulanger P, Letellier L (1996). FhuA, a transporter of the *Escherichia coli* outer membrane, is converted into a channel upon binding of bacteriophage T5. EMBO J..

[39] Shin H (2012). Receptor diversity and host interaction of bacteriophages infecting Salmonella enterica serovar Typhimurium. PLoS ONE.

[40] Labrie SJ, Samson JE, Moineau S (2010). Bacteriophage resistance mechanisms. Nat. Rev. Microbiol..

[41] Pawelek PD (2006). Structure of TonB in complex with FhuA, *E. coli* outer membrane receptor. Science.

[42] Yang JH, Bening SC, Collins JJ (2017). Antibiotic efficacy—context matters. Curr. Opin. Microbiol..

[43] McHugh JP (2003). Global iron-dependent gene regulation in *Escherichia coli*. A new mechanism for iron homeostasis. J. Biol. Chem..

[44] Meng X (2016). Virulence characteristics of extraintestinal pathogenic *Escherichia coli* deletion of gene encoding the outer membrane protein X. J. Vet. Med. Sci..

[45] Atkinson S, Williams P (2016). Yersinia virulence factors—a sophisticated arsenal for combating host defences. F1000Research..

[46] Aabo S, Rasmussen OF, Roseen L, Sørensen PD, Olsen JE (1993). *Salmonella* identification by the polymerase chain reaction. Mol. Cell. Probes.

[47] Holloway BW (1955). Genetic recombination in *Pseudomonas aeruginosa*. Microbiology.

[48] Datsenko KA, Wanner BL (2000). One-step inactivation of chromosomal genes in *Escherichia coli* K-12 using PCR products. Proc. Natl. Acad. Sci..

[49] 49Grimon, D., Gerstmans, H., Briers, Y. & Lavigne, R. (2018). POLYNUCLEOTIDE SHUFFLING METHOD. WO2018114980. Available at: https://be.espacenet.com/publicationDetails/biblio?FT=D&date=20180628&DB=EPODOC&locale=nl_BE&CC=WO&NR=2018114980A1&KC=A1&ND=4

[50] Gerstmans H (2020). A VersaTile-driven platform for rapid hit-to-lead development of engineered lysins. Sci. Adv..

[51] Lavigne R, Briers Y, Hertveldt K, Robben J, Volckaert G (2004). Identification and characterization of a highly thermostable bacteriophage lysozyme. Cell. Mol. Life Sci..

[52] Briers Y, Lavigne R, Volckaert G, Hertveldt K (2007). A standardized approach for accurate quantification of murein hydrolase activity in high-throughput assays. J. Biochem. Biophys. Methods.

[53] Database resources of the National Center for Biotechnology Information. *Nucleic Acids Res.* **41**, D8–D20, doi:10.1093/nar/gks1189 (2013). 10.1093/nar/gks1189 PMC3531099 23193264

[54] Sievers, F. *et al.* Fast, scalable generation of high‐quality protein multiple sequence alignments using Clustal Omega. *Mol. Syst. Biol.* **7**, doi:10.1038/msb.2011.75 (2011). 10.1038/msb.2011.75 PMC3261699 21988835

[55] Waterhouse AM, Procter JB, Martin DMA, Clamp M, Barton GJ (2009). Jalview Version 2—a multiple sequence alignment editor and analysis workbench. Bioinformatics.

