# Supplementary material for: Exploiting phage receptor binding proteins to enable endolysins to kill Gram-negative bacteria
Source: Sci Rep. 2020 Jul 21;10:12087. doi: 10.1038/s41598-020-68983-3 (PMC7374709; doi:10.1038/s41598-020-68983-3)
Supplement: Supplementary file 1 — Supplementary file1 [file 41598_2020_68983_MOESM1_ESM.pdf]

Supplementary material for:

Exploiting phage receptor binding proteins to enable endolysins to kill Gram-negative bacteria

Athina Zampara[1], Martine C. Holst Sørensen[1], Dennis Grimon[2], Fabio Antenucci[1], Amira Vitt[1], Valeria Bortolaia[3], Yves Briers[2] and Lone Brøndsted[1*]

[1]Department of Veterinary and Animal Sciences, University of Copenhagen, Stigbøjlen 4, 1870 Frederiksberg C, Denmark

[2]Department of Biotechnology, Ghent University, Valentin Vaerwyckweg 1, 9000 Gent, Belgium

[3]National Food Institute, Technical University of Denmark, WHO Collaborating Center for Antimicrobial Resistance in Food borne Pathogens and Genomics and European Union Reference Laboratory for Antimicrobial Resistance, Kemitorvet 204, 2800 Kongens Lyngby, Denmark

*Address correspondence to Lone Brøndsted: lobr@sund.ku.dk

THIS SUPPLEMETARY FILES CONTAINS:

SUPPLEMENTARY FIGURES **2**

Figure S1: Workflow for selecting Innolysins with high antibacterial efficiency. 2

Figure S2: Muralytic assay used to eliminate enzymatically inactive Innolysins. 3

Figure S3: Growth inhibition of *E. coli* after treatment with Innolysins. 4

SUPPLEMENTARY TABLES **5**

Table S1: Matrix showing percent identity of BL21 FhuA homologs. 5

Table S2: Endolysins used for engineering the Innolysins Ec. 6

Table S3: Initial screening of growth inhibition of *E. coli* after Innolysin treatment. 8

Table S4: Modular composition of 48 selected Innolysins showing the highest inhibitory effect during the initial high-throughput screening. 9

Table S5: *E. coli* strains resistant to third-generation cephalosporins used for testing antibacterial activity of Innolysin Ec21 11

Table S6: Primers used for PCR amplification. 12

METHOD USED FOR LIBRARY CONSTRUCTION OF 228 NOVEL INNOLYSINS **15**

SUPPLEMENTARY FIGURES

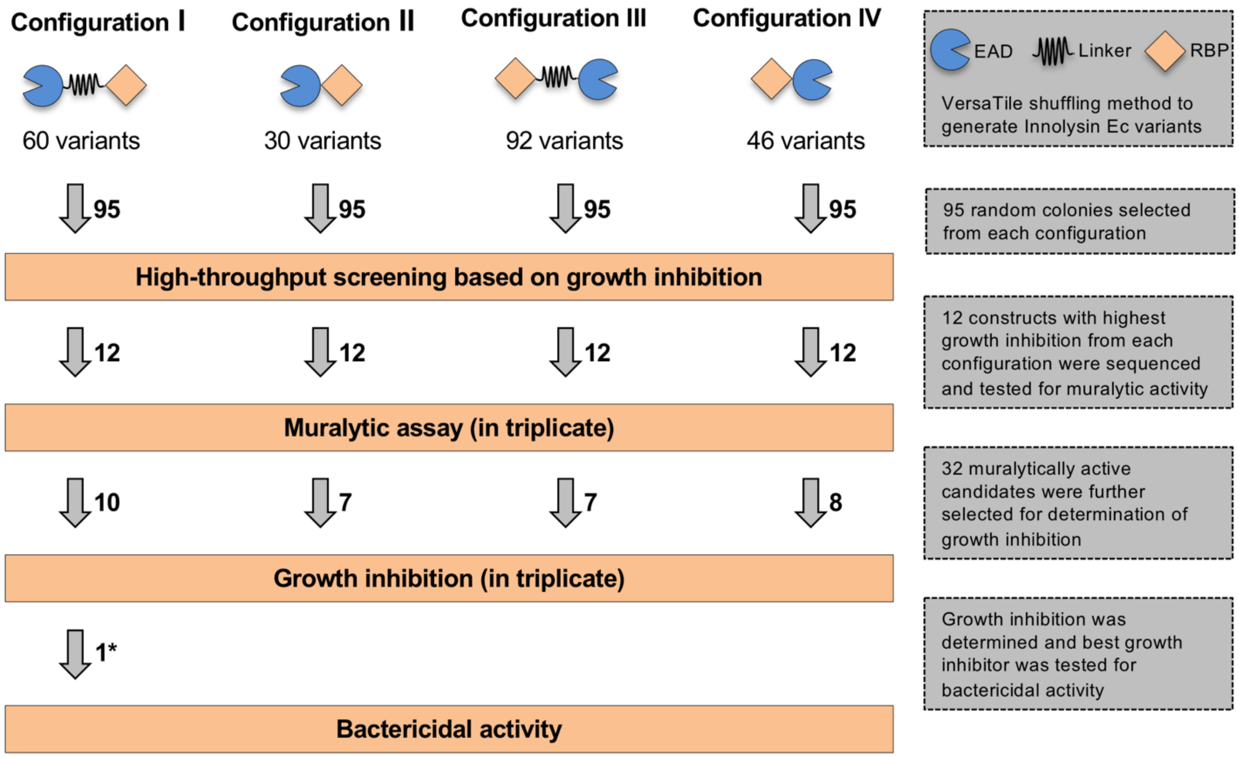

Figure S1: Workflow for selecting Innolysins with high antibacterial efficiency. Versatile approach was used to construct Innolysins composed of one out of 23 unique endolysins (supplementary Table S2). Enzymatic active domains of endolysins were fused with phage T5 receptor binding protein (RBP), Pb5, or the binding domain of Pb5 ($Pb5_{1\text{-}488}$) in different configurations. EAD: Enzymatically active domain; Linker: Linker 1 (six amino acids) or Linker 2 (fourteen amino acids); RBP: Pb5 or binding domain of Pb5 ($Pb5_{1\text{-}488}$). In total 228 novel Innolysins were constructed. 95 colonies of each configuration were randomly selected and screened for growth inhibition of *E. coli* BL21. The 12 best performing candidates in terms of growth inhibition were selected from each configuration based on high-throughput screening. To eliminate the muralytically inactive Innolysin candidates, a muralytic assay was conducted and 32 constructs were withheld. The growth inhibition activities of cleared lysates of the muralytically active

Innolysins (n=32) were further determined in triplicate against *E. coli* BL21. The Innolysin with the highest growth inhibition activity (Ec21) was purified and its antibacterial activity and spectrum were determined. * Innolysin Ec21.

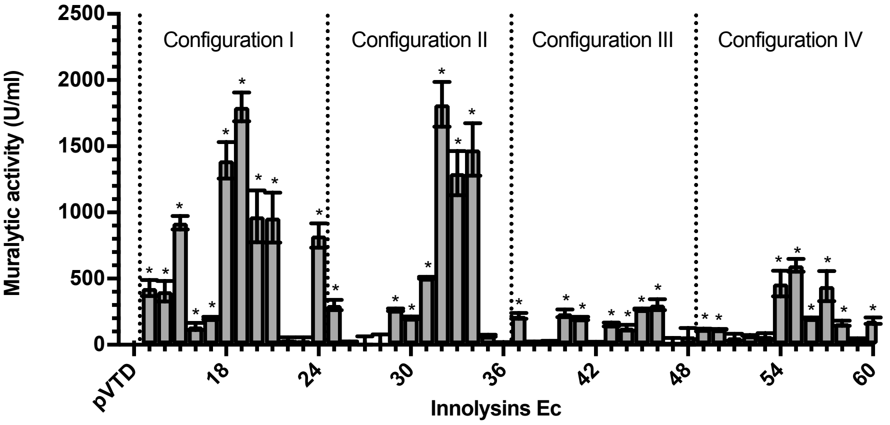

Figure S2: Muralytic assay used to eliminate enzymatically inactive Innolysins. The 48 selected Innolysins with different configurations (Table S4) were screened for muralytic activities on outer membrane permeabilized *P. aeruginosa* substrate. Cleared lysates were tested in triplicate and compared to the activity of cleared lysate of cells carrying the empty vector pVTD (negative control). Innolysin enzymatic activities were reported in units per milliliter lysate (U/ml) but not normalized for product expression yield * Significant muralytic activity compared to the negative control at $P < 0.05$.

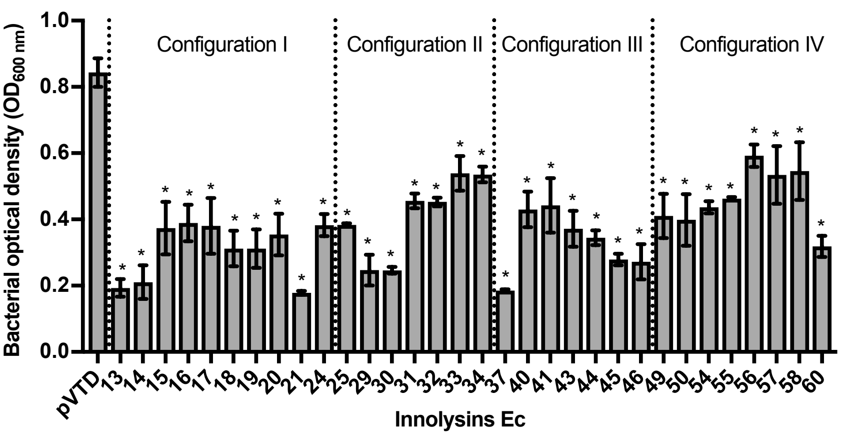

Figure S3: Growth inhibition of *E. coli* after treatment with Innolysins. Innolysins with muralytic activity (Fig. S2) were retested in triplicate for the ability to significantly inhibit the growth of *E. coli* BL21. Cells were mixed with cleared lysates of cells expressing the proteins and turbidity of the suspensions was measured spectophotometrically after incubation for 18 hours at 37°C and compared to the negative control (cleared lysate of cells carrying the empty vector pVTD). * Significant bacterial growth inhibition at $P < 0.05$.

SUPPLEMENTARY TABLES

Table S1: Matrix showing percent identity of BL21 FhuA homologs. Percent identity between *E. coli* BL21 FhuA and homologous proteins was calculated by using the sequence alignment tool Clustal Omega. Full-length FhuA protein sequences of *E. coli* BL21 (WP_000124402.1), *E. coli* ECOR4 (locus tag ESC_AA8779AA_AS_03052, PRJEB2879) and *Shigella sonnei* (WP_094317049.1) were used for constructing the identity matrix. Furthermore, homologous proteins of *Pseudomonas aeruginosa* PAO1, including FiuA (NP_249161.1), FoxA (NP_251156.1), FptA (NP_252911.1), FpvA (NP_251088.1) and FpvB (NP_252857.1), were compared to *E. coli* BL21 FhuA (WP_000124402.1).

Strains	FhuA *E. c.* BL21	FhuA *E. c.* ECOR4	FhuA *S. sonnei*	FiuA *P.a.* PAO1	FoxA *P.a.* PAO1	FptA *P.a.* PAO1	FpvA *P.a.* PAO1	FpvB *P.a.* PAO1
FhuA *E. c.* BL21	100.00	99.87	99.60	36.53	38.52	22.61	23.02	23.78
FhuA *E. c.* ECOR4	99.87	100.00	99.73	36.57	38.55	22.54	22.95	23.71
FhuA *S. sonnei*	99.60	99.73	100.00	36.57	38.55	22.54	22.95	23.71
FiuA *P.a.* PAO1	36.53	36.57	36.57	100.00	39.26	23.17	22.67	23.54
FoxA *P.a.* PAO1	38.52	38.55	38.55	39.26	100.00	24.15	23.53	25.97
FptA *P.a.* PAO1	22.61	22.54	22.54	23.17	24.15	100.00	33.95	35.81
FpvA *P.a.* PAO1	23.02	22.95	22.95	22.67	23.53	33.95	100.00	39.90
FpvB *P.a.* PAO1	23.78	23.71	23.71	23.54	25.97	35.81	39.90	100.00

Table S2: Endolysins used for engineering the Innolysins Ec.

**Endolysins**<br>Accession number	**Amino acids**	**Origin**	**Pfam**	**Isoelectric point**	**Config.**
**PsP3gp10**<br>NP_958065.1	165	*Salmonella enterica* phage PsP3	Phage lysozyme CL0037	9.47	I-IV
**P2gp09***<br>NP_046765.1	165	*Escherichia coli* phage P2	Phage lysozyme CL0037	9.79	I-IV
**K11gp3.5**<br>AAX62800	151	*Klebsiella pneumoniae* phage K11	Amidase_2 N-acetylmuramoyl-L-alanine amidase	8,48	I-IV
**CR8gp3.5**<br>YP_009004176	152	*Citrobacter* phage CR8 -	Amidase_2 N-acetylmuramoyl-L-alanine amidase	7,09	I-IV
**BcepC6Bgp22**<br>YP_024942.1	164	*Burkholderia cepacia* phage BcepC6B	Phage lysozyme CL0037	9,16	I-IV
**LysEC8**<br>YP_007348465	166	*Escherichia* phage phAPEC8	Phage lysozyme CL0037	9,16	I-IV
**KZ144-EAD***<br>NP_803710.1	177	*Pseudomonas* phage phiKZ	Transglycosylase SLT domain CL0037	9,42	I-IV
**EL188-EAD***<br>YP_418221	177	*Pseudomonas* phage EL188	-	8,47	I-IV
**OBPgp279-EAD**<br>YP_004958186	201	*Pseudomonas fluorescens* phage OBP	COG3179	7,66	I-IV
**LysAci7**<br>No accession number	193	-	-	9,64	I-IV
**Phage2b.1bgp019**<br>Unpublished	187	-	DUF3380 Protein of unknown function	8,8	I-IV
**XccBgp35**<br>Unpublished	189	-	-	9,3	I-IV
**Phage003/011gp040**	137	-	Peptidase_M15_4	7,97	I-IV

Unpublished			D-alanyl-D-alanine carboxypeptidase		
**Vpept*** YP_007518361	141	*Vibrio* phage VvAW1	D-alanyl-D-alanine carboxypeptidase cl00813	8,03	I-IV
**KZgp181*** AAL83082.1	163	*Pseudomonas* phage phiKZ	Transglycosylase SLT domain	6,84	I-IV
**201Phi2-1gp229-EAD*** YP_001956952.1	184	*Pseudomonas chlororaphis* phage 201phi2-1	(DUF3380 Protein of unknown function)	8,41	III-IV
**PVP-SE1gp146-EAD*** YP_004893952	184	*Salmonella* phage PVP-SE1	Chitinase class I CL0037	8,41	III-IV
**KMVgp36C*** NP_877475.1	162	*Pseudomonas* phage phiKMV	Lysozyme	5,22	III-IV
**KP32gp15** YP_003347533.1	152	*Klebsiella pneumoniae* phages KP32	Amidase_2 N-acetylmuramoyl-L-alanine amidase	7,79	III-IV
**BcepC6Bgp16*** YP_024936.1	137	*Burkholderia virus* BcepC6B	Transglycosylase SLT domain	8,12	III-IV
**OBPgp276** YP_004958183.1	176	*Pseudomonas fluorescens* phage OBP	Glyco_hydro_19	9,72	III-IV
**ORF007_55gp7_CHAP*** YP_240484.1	158	*Staphylococcus aureus* phage	Amidase	5,34	III-IV
**LUZ24gp67*** YP_001671940.1	133	*Pseudomonas aeruginosa* phage LUZ24	Lysozyme	8	III-IV

*Endolysins that are not components of the 48 Innolysins selected after the initial high-throughput screening.

Table S3: Initial screening of growth inhibition of *E. coli* after Innolysin treatment. *E. coli* BL21 was treated with cleared lysates of cells expressing Innolysins at 37ºC and optical densities were measured after 18 hours of incubation. The different configurations of Innolysins are described in Figure S1.

Optical density (OD_{600}) after treatment with Innolysins	Number of Innolysins in category				
	Configuration I	Configuration II	Configuration III	Configuration IV	Empty vector (pVTD)
0-0.35	28/95 (30%)	16/95 (17%)	7/95 (8%)	12/95 (13%)	0/4 (0%)
0.35-0.75	65/95 (68%)	44/95 (46%)	44/95 (46%)	66/95 (69%)	0/4 (0%)
0.75-1.00	2/95 (2%)	35/95 (37%)	44/95 (46%)	17/95 (18%)	4/4 (100%)

Table S4: Modular composition of 48 selected Innolysins showing the highest inhibitory effect during the initial high-throughput screening. All constructs were fused with a His-tag in the C-terminus and the 12 best-growth inhibitors were selected per each configuration for further testing the antibacterial activity.

Constructs	Position 1	Position 2	Position 3	Size (kDa)
**Configuration I**				
**13**	XccBgp35	Linker 2	Pb5	95,7
**14**	OBPgp279	Linker 1	Pb5	96,7
**15**	LysEC8	Linker 1	Pb5	92,4
**16**	PsP3gp10	Linker 2	$Pb5_{1\text{-}488}$	75,1
**17**	OBPgp279	Linker 2	Pb5	93,8
**18**	Phage003/011gp040	Linker 1	Pb5	89,3
**19**	LysEC8	Linker 1	Pb5	92,4
**20**	LysEC8	Linker 2	Pb5	92,9
**21**	LysEC8	Linker 2	$Pb5_{1\text{-}488}$	75,1
**22***	LysAci7	Linker 2	Pb5	95,9
**23***	PsP3gp10	Linker 1	$Pb5_{1\text{-}488}$	74,6
**24**	LysEC8	Linker 2	Pb5	92,9
**Configuration II**				
**25**	Phage003/011gp040	$Pb5_{1\text{-}488}$	His-tag	71,1
**26***	CR8gp3.5	Pb5	His-tag	91,1
**27***	PsP3gp10	Pb5	His-tag	92,0
**28***	KP32gp15	Pb5	His-tag	90,8
**29**	PsP3gp10	$Pb5_{1\text{-}488}$	His-tag	74,2
**30**	Phage2b.1bgp019	$Pb5_{1\text{-}488}$	His-tag	76,2
**31**	OBPgp279	Pb5	His-tag	96,3
**32**	LysEC8	$Pb5_{1\text{-}488}$	His-tag	74,2
**33**	LysEC8	Pb5	His-tag	92,0
**34**	LysEC8	Pb5	His-tag	92,0
**35***	OBPgp279	$Pb5_{1\text{-}488}$	His-tag	78,5
**36***	BcepC6Bgp22	$Pb5_{1\text{-}488}$	His-tag	73,4
**Configuration III**				
**37**	$Pb5_{1\text{-}488}$	Linker 2	Phage2b.1bgp019	77,1
**38***	$Pb5_{1\text{-}488}$	Linker 2	BcepC6Bgp22	74,3
**39***	Pb5	Linker 2	XccBgp35	95,7
**40**	Pb5	Linker 2	Phage2b.1bgp019	94,9
**41**	Pb5	Linker 2	Phage2b.1bgp019	94,9
**42***	$Pb5_{1\text{-}488}$	Linker 2	K11gp3.5/PsP3gp10	91,8
**43**	Pb5	Linker 2	Phage2b.1bgp019	94,9

**44**	Pb5	Linker 1	LysEC8	92,4
**45**	$Pb5_{1-488}$	Linker 2	OBPgp279	79,5
**46**	Pb5	Linker 1	XccBgp35	95,2
**47***	$Pb5_{1-488}$	Linker 2	CR8gp3.5	74,2
**48***	$Pb5_{1-488}$	Linker 2	PsP3gp10	75,1
**Configuration IV**				
**49**	$Pb5_{1-488}$	Phage2b.1bgp019	His-tag	76,2
**50**	$Pb5_{1-488}$	Phage2b.1bgp019	His-tag	76,2
**51***	Pb5	Phage2b.1bgp019	His-tag	94,0
**52***	$Pb5_{1-488}$	Phage2b.1bgp019	His-tag	76,2
**53***	$Pb5_{1-488}$	BcepC6Bgp22	His-tag	73,4
**54**	Pb5	K11gp3.5	His-tag	90,1
**55**	$Pb5_{1-488}$	Phage003/011gp040	His-tag	71,1
**56**	Pb5	Phage2b.1bgp019	His-tag	94,0
**57**	Pb5	PsP3gp10	His-tag	92,0
**58**	Pb5	BcepC6Bgp22	His-tag	91,2
**59***	Pb5	Phage2b.1bgp019	His-tag	94,0
**60**	Pb5	Phage2b.1bgp019	His-tag	94,0

*Constructs that did not display significant muralytic activity.

Table S5: ***E. coli*** **strains resistant to third-generation cephalosporins used for testing antibacterial activity of Innolysin Ec21.** The table includes the original name and the European Nucleotide Archive (ENA) reference where the raw sequencing data may be found. Furthermore, the source that strains were isolated from and the ST, Sequence Type as defined by Multi Locus Sequence Typing (MLST) are described. All strains have an upregulated expression of chromosomal ampC gene, confering resistance to third-generation cephalosporins.

**Short name**	**Original name**	**ENA reference (sample name)**	**Source**	**ST-type**
**702**	DTU2016_702_PRJ1055_Escherichia_coli_528_15097558_1	ERS1229481	Pig caecal	ST-453
**708**	DTU2016_708_PRJ1055_Escherichia_coli_528_15083216_1	ERS1229487	Pig caecal	ST-20
**723**	DTU2016_723_PRJ1055_Escherichia_coli_528_15108571_1	ERS1229502	Pig caecal	ST-88
**724**	DTU2016_724_PRJ1055_Escherichia_coli_528_15108578_1	ERS1229503	Pig caecal	ST-1800
**728**	DTU2016_728_PRJ1055_Escherichia_coli_528_15084086_1	ERS1229507	Pig caecal	ST-23
**770**	DTU2016_770_PRJ1055_Escherichia_coli_903_15116019_1	ERS1229549	Chicken meat	ST-6206

Table S6: Primers used for PCR amplification. To engineer recombinant proteins, *oad* gene encoding phage T5 Pb5 (YP_006985.1) and *lys* gene encoding phage T5 endolysin (YP_006868.1) were used for amplification. Linkers L1: AGAGAG and L2: GAGAGAGAGAGAGA were created as primer cassettes. SapI sequences are boxed and they were used for cloning in pVTE, while the BsaI sequences (grey) were used for shuffling. Underlined sequences indicate the position of the building block in the assembled sequence.

Primers	Sequence	Size
lys position 1 Frw	TGTGCTCTTCCAGAGGTCTCACCATGAGTTTTAAATTTGGTAAAAATAGCGAAAAAC	57
lys position 1 Rvs	AGAGCTCTTCACTTGGTCTCAGCACCAACTAGTTCGACATGACCGCCATC	50
lys position 2 Frw	TGTGCTCTTCCAGAGGTCTCGGTGCTAGTTTTAAATTTGGTAAAAATAGCGAAAAAC	57
lys position 2 Rvs	AGAGCTCTTCACTTGGTCTCGCCTGCAACTAGTTCGACATGACCGCCATC	50
lys position 3 Frw	TGTGCTCTTCCAGAGGTCTCGCAGGCAGTTTTAAATTTGGTAAAAATAGCGAAAAAC	57
lys position 3 Rvs	TGTGCTCTTCACTTGGTCTCGCTTCCAACTAGTTCGACATGACCGCCATC	50
oad position 1 Frw	TGTGCTCTTCAAGAGGTCTCACCATGAGTTTTTTCGCTGGCAAGC	45
oad position 1 Rvs	TGTGCTCTTCACTTGGTCTCTGCACCGGTAAGGCGTTGAAT	58

	TATAAGTTTAATATTAG	
oad position 2 Frw	TGTGCTCTTCAAGAGGTCTCGGTGCTAGTTTTTCGCTGGC AAGC	45
oad position 2 Rvs	TGTGCTCTTCACTTGGTCTCGCCTGCGGTAAGGCGTTGAAT TATAAGTTTAATATTAG	58
oad position 3 Frw	TGTGCTCTTCTAGAGGTCTCGCAGGCAGTTTTTCGCTGGC AAGC	45
oad position 3 Rvs	AGAGCTCTTCACTTGGTCTCGCTTCCGGTAAGGCGTTGAA TTATAAGTTTAATATTAG	58
oad_{1-1464} position 1 Frw	TGTGCTCTTCAAGAGGTCTCACCATGAGTTTTTCGCTGGC AAGC	45
oad_{1-1464} position 1 Rvs	ACAGCTCTTCACTTGGTCTCAGCACCATCTAACCCCACAG TACCAAC	47
oad_{1-1464} position 2 Frw	TGTGCTCTTCAAGAGGTCTCGGTGCTAGTTTTTCGCTGGC AAGC	45
oad_{1-1464} position 2 Rvs	TGTGCTCTTCACTTGGTCTCGCCTGCATCTAACCCCACAGT ACCAAC	47
oad_{1-1464} position 3 Frw	TGTGCTCTTCTAGAGGTCTCGCAGGCAGTTTTTCGCTGGC AAGC	45
oad_{1-1464} position 3 Rvs	TGTGCTCTTCACTTGGTCTCGCTTCCATCTAACCCCACAGT ACCAAC	47
Linker 1 Frw	GCGGGTGCGGGTGCGGGT	18
Linker 1 Rvs	ACCCGCACCCGCACCCGC	18

Linker 2 Frw	AGCAAGCTTGGTCTCAGTGCAGGCGCGGGCGCGGGCGCG GGCGCGGGCGCGGGCGCGGGCGCG	63
Linker 2 Rvs	AGCTCTAGAGGTCTCACCTGCCGCGCCCGCGCCCGCGCCC GCGCCCGCGCCCGCGCCCGCGCC	63

METHOD USED FOR LIBRARY CONSTRUCTION OF 228 NOVEL INNOLYSINS

Here, we aimed to enhance the antibacterial activity of Innolysins by fusing different endolysins with either the whole Pb5 or the binding domain of Pb5 ($Pb5_{1-488}$). For this purpose, we used an endolysin library consisting of 23 endolysins presenting diverse enzymatic activities and architectures, with either globular or modular organisation (Table S2). Only the enzymatically active domains of the endolysins (EAD) were used for engineering. To determine whether a specific configuration of Innolysins was optimal for antimicrobial activity, a systematic setup of hybrid construction was conducted in four configurations (Fig. S1). Constructs with configuration I and II were composed of EADs in N-terminus and RBPs in the C-terminus, whereas constructs with configuration III and IV comprised the domains in opposite direction, with the EADs located in the C-terminus instead. Fiffteen distinct EADs were used for configuration I and II and 23 EADs for configuration III-IV (Table S4). Additionaly, in configuration II and IV domains were fused directly, while in configuration I and III the domains were joined with either linker L1 comprising six amino acids or linker L2 comprising 14 amino acids. In practice, different domain candidates were recombined in a combinatorial library in a predesigned order by using the VersaTile technique. For instance, for configuration I, we mixed the 15 different EAD domains for position 1, the two linkers for position 2 and Pb5 with $Pb5_{1-488}$ for position 3, which results for this configuration in a modular library of 60 (15*2*2) different variants. We randomly selected 95 single colony transformants from this combinatorial library, with every variant having the same overall architecture but differing in composition. The same approach was used for the three other possible configurations. In total, 228 novel Innolysins were constructed and 380 Innolysins were selected equally from four configuration libraries and screened in high-throughput by a growth inhibition assay on BL21 cells as described in Material and Methods.
